# Resveratrol Modulates Mitochondria Dynamics in Replicative Senescent Yeast Cells

**DOI:** 10.1371/journal.pone.0104345

**Published:** 2014-08-06

**Authors:** I-Hua Wang, Hsin-Yi Chen, Yu-Han Wang, Ko-Wei Chang, Ying-Chieh Chen, Chuang-Rung Chang

**Affiliations:** 1 Institute of Biotechnology, National Tsing Hua University, Hsin Chu City, Taiwan; 2 Department of Medical Science, National Tsing Hua University, Hsin Chu City, Taiwan; Taipei Medical University, Taiwan

## Abstract

Mitochondria form a reticulum network dynamically fuse and divide in the cell. The balance between mitochondria fusion and fission is correlated to the shape, activity and integrity of these pivotal organelles. Resveratrol is a polyphenol antioxidant that can extend life span in yeast and worm. This study examined mitochondria dynamics in replicative senescent yeast cells as well as the effects of resveratrol on mitochondria fusion and fission. Collecting cells by biotin-streptavidin sorting method revealed that majority of the replicative senescent cells bear fragmented mitochondrial network, indicating mitochondria dynamics favors fission. Resveratrol treatment resulted in a reduction in the ratio of senescent yeast cells with fragmented mitochondria. The readjustment of mitochondria dynamics induced by resveratrol likely derives from altered expression profiles of fusion and fission genes. Our results demonstrate that resveratrol serves not only as an antioxidant, but also a compound that can mitigate mitochondria fragmentation in replicative senescent yeast cells.

## Introduction

Mitochondria are responsible for ATP synthesis, calcium buffering, and apoptosis. Numerous studies have identified them as organelles pivotal to processes of cell signaling, proliferation, aging, disease, and death [1]–[3]. Unlike static organelles, mitochondria form a reticulum dynamically fuse and divide in the cell. Continuous fusion and fission shape the morphology of mitochondrial network and play a key role in maintaining the integrity of the mitochondria. Excessive fusion leads to a hyperfused/elongated network, and extra fission causes fragmented one [4]. The balance between fusion and fission corresponds to environmental stress signals and the functional versatility of the mitochondria [5]–[7].

The mechanisms involved in mitochondrial fusion and fission were evolutionarily conserved from yeast to mammals [8]. In *Saccharomyces cerevisiae*, fusion and fission are antagonistically regulated by Fzo1 and Dnm1 according to the physiological conditions [9]. Fzo1 is a large GTPase mitofusin family protein responsible for the fusion of the mitochondrial outer membrane [10]–[12]. Fzo1 is joined with Mgm1 and Ugo1 to complete mitochondrial fusion [12], [13]. Dnm1 is also a large GTPase dynamin-related protein recruited in the fission complexes with Fis1, Mdv1 and Caf4 at the mitochondrial outer membrane to promote division [14]–[17].

Replicative and chronological aging models of budding yeast have great contributions for identifying molecular mechanisms related to aging. Replicative life span is determined by the number of budding cycles a mother cell went through. Chronological life span refers to the time period a non-budding cell can sustained [18]. Resveratrol is a polyphenol compound originally identified in plants. Studies have shown that it can extend the life span of both yeast and *Caenorhabditis elegans* by mimicking the conditions of restricted caloric intake that depend on Sir2 [19]–[22]. Resveratrol also shows antioxidant properties as it directly scavenges free radicals and promotes the function of enzymatic antioxidants in cells. Resveratrol has also been shown to improve mitochondrial activity and stimulate autophagy through the activation of the AMPK pathways involving PGC-1 [23]–[25].

The importance of mitochondria in pathological aging cannot be overstated. Mitochondrial integrity is a crucial factor in aging and age-related diseases [26]. Irregular mitochondria dynamics has been implicated in many neurodegenerative diseases, such as Parkinson’s and Alzheimer’s diseases [27], [28]. The morphology, function, and homeostasis of mitochondria relate to the strict regulation of fusion and fission processes, the dynamics of which are thought to adjust according to physiological conditions in senescence. This paper focus on elucidating the status of mitochondria dynamics in replicative senescent yeast cells and clarifying whether resveratrol influences the processes of fusion and fission in these cells. Our results demonstrate that mitochondria dynamics in senescent cells differs from that of young cells, and resveratrol alters the balance of mitochondrial fusion and fission in replicative senescent yeast cells.

## Materials and Methods

### Strains and culturing conditions

*Saccharomyces cerevisiae* strain W303-1a (*MATa, his3Δ, leu2Δ, trp1Δ, ura3Δ*) was used as the wild type parental strain. Mutant strains including *dnm1* and *fzo1* were derived from W303-1a by replacing genomic loci with hygromycin B phosphotransferase (HPH) cassettes. HA-tagged *DNM1* strain was constructed by direct gene replacement of HA cassette at the end of *DNM1*. Liquid YPD (1% yeast extract, 2% peptone, 2% dextrose in H_2_O) and synthetic complete (SC) growth medium (0.17% yeast nitrogen base without amino acids and ammonium sulfate, 0.5% ammonium sulfate) were used for yeast cell cultures at 30°C. Cell treatment involved a final concentration of 30 µM Resveratrol (Cat. R5010, SIGMA, U.S.A.), which was added to the medium 14 hours after biotin labeling.

### Sorting senescent cells

All cell sorting was performed in accordance with the protocols outlined in previous studies [29], [30]. Cells were inoculated in synthetic complete (SC) growth medium overnight before being sub-cultured until mid-log phase (O.D. = 0.3). Cells were harvested and labeled using EZ-Link Sulfo-NHS-LC-biotin (Cat.21327, Thermo Scientific, U.S.A.). Biotin labelled cells were then inoculated in 50 ml SC medium and cultured at 30°C for at least 48 hours. Streptavidin magnetic beads (Cat.88817, Thermo Scientific, U.S.A.) were added to interact with biotin-labelled cells, prior to sorting with magnets.

### Fluorescence microscopy

Cell samples were fixed in 3.7% formaldehyde for 1 hour at 30°C. A fluorescence microscope (Axioskop 2 mot plus; Carl Zeiss, Germany) was used to visualize yeast cells containing pVT100-mtGFP [31]. Images were processed and analyzed using software Axiovision 4.6. The classification of the mitochondrial network morphology followed the criteria of previous reports and judged by at least two individuals [8], [32], [33]. Solophenyl Flavine 7GFE 500% (Town End (Leeds) plc, UK) was used to stain the cell wall and bud scars [34].

### DNA extraction and RNA preparation

Cells were ruptured using acid-wash glass beads with a lysis buffer (2% Triton-100, 100 mM NaCl, 10 mM pH 8.0 Tris-Cl, 10 mM EDTA, 1% SDS). Genomic DNA along with mitochondrial genome were extracted using phenol/chloroform. To extract RNA from the yeast, the ZR Fungal/Bacterial RNA MiniPrepTm kit (Cat. R2014, Zymo Research, U.S.A.) was used. DNA and RNA concentrations were measured by NanoDrop 2000 spectrophotometer (Thermo Scientific, U.S.A.).

### Reverse transcription and quantitative polymerase chain reaction

The Deoxy+ HiSpec Reverse Transcriptase Kit (Yeastern Biotech, Taiwan) was used for reverse transcription. The thermal cycler ABI PRISM 7500 or ABI StepOne Plus Real-Time PCR System were used in conjunction with the MicroAmp Optical 8-Tube Strip, 0.2 ml (Cat. 4316567) and Roche FastStart Universal SYBR Green Master (Cat. 0453528601) to perform quantitative PCR. The results were analyzed by ABI software v2.0.5. Fold changes were determined using the ΔΔCT method. A subunit for signal recognition particle gene *SCR1* was used as an endogenous control. All sets of primer sequences used for nuclear genes and the mitochondrial genome quantification in this study will be provided upon request.

### Western blot

Yeast proteins were harvested by glass beads grinding in PBS buffer with vigorous vortex. Total proteins were subjected to electrophoresis in SDS-PAGE gel and transferred to nitrocellulose membrane. Anti-HA (Cat. 05904, Millipore, U.S.A.) was used to detect Dnm1-HA. Relative Dnm1 levels were determined by Odyssey Infrared Imaging Systems (LI-COR Bioscience, U.S.A.), and normalized based on actin levels.

### Mitochondrial membrane potential, Superoxide level, Annexin V staining and Flow cytometry

Mitochondrial membrane potential was measured by either Rhodamine 123 (Cat. R8004, SIGMA, U.S.A.) or DiOC_6_ (3) (Cat. D273, Life Technology, U.S.A.) followed the instructions provided by the companies. MitoSOX Red (Cat. M36008, Molecular probe, U.S.A.) and Dihydroethidium (Cat. D7008, SIGMA, U.S.A.) were used as mitochondrial and intracellular superoxide indicator. Annexin V-FITC Apoptosis Detection Kit from Strong Biotech Corporation (Taiwan) were used to determine early apoptosis. The samples were analyzed by flow cytometer FACSCalibur and BD Accuri C6 (BD Bioscience, San Jose, CA) followed by the protocol provided by the company. For each sample, 10,000 cells were analyzed. The percentage of positive stained cell numbers were determined under the comparison of stained and control cells by Cellquest software from BD Bioscience.

### Statistical analysis

All experiments were performed at least three times. For the classification of mitochondrial morphology, at least one hundred cells were examined in each trial. Data are expressed as mean values with standard deviation (SD). All error bars in the figures were based on standard error unless specifically mentioned. Significance was determined using two-tailed unpaired Student’s t tests.

## Results

**Figure 1:**
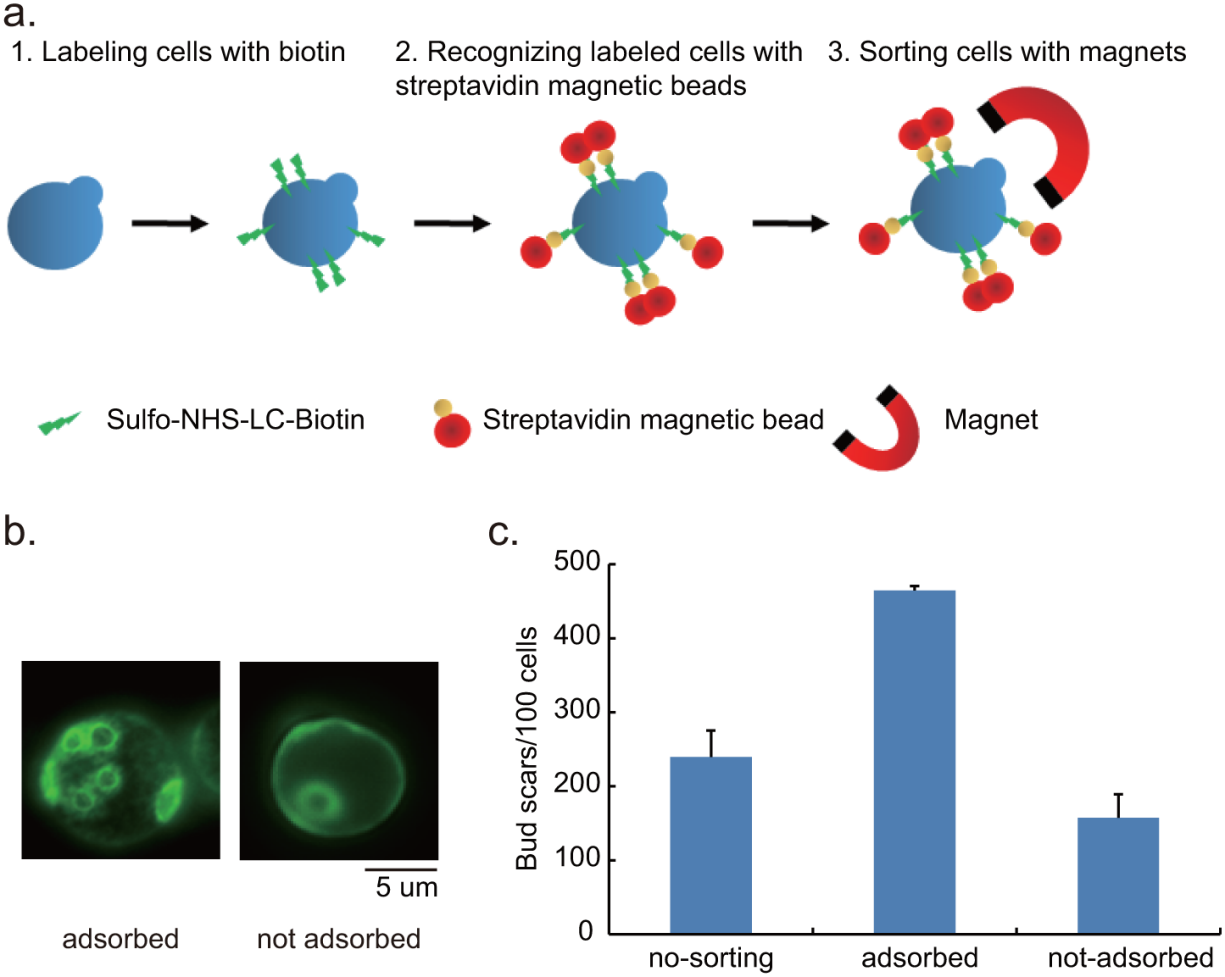
Sorting and validating replicative senescent yeast cells. **a**. Biotin-Streptavidin method for sorting senescent cells was performed as depicted. **b**. A comparison of bud scars on adsorbed and not adsorbed yeast cells stained by Solophenyl Flavine 7GFE 500%. **c**. Yeast cells were cultured for 48 hours and then divided into three groups: no-sorting, adsorbed by magnets and those not adsorbed by magnets. The bud scars for a hundred cells were counted. The average number of bud scars on the adsorbed cells was approximately three times the figures obtained for not-adsorbed cells.

Collecting replicative senescent yeast cells can be achieved by using the streptavidin-biotin interaction to sort out mother cells in a population [29], [30], or by using the mother enrichment program (MEP) [35]. This study used the streptavidin-biotin method for the preparation of senescent cells. We added Sulfo-NHS-LC-biotin at the initiation of culturing to label mother cells, and asymmetrical division ensured that a greater proportion would be apportioned to mother cells than to daughter cells [18]. Following growth for hours in the liquid medium, magnetic streptavidin beads were used to isolate biotin-labeled cells from newly budded daughter cells by magnets. (Fig. 1a). We selected this method because it does not disrupt sumoylation-related gene *UBC9* as MEP procedure required. Sumoylation has been shown to play a role in mitochondria dynamics [36]–[38].

### Replicative senescent cells are larger with more bud scars

After labeling cells in the log phase of growth, the residual biotin was washed out and labeled cells were cultured for at least 48 hours. Cell size in budding yeast is known to increase gradually with culturing time; therefore, we measured the size of the cells adsorbed by magnets to verify that they went through more divisions. Our results showed that the average cell size of the log phase cells was smaller than those in samples obtained 10, 22, and 48 hours after labeling. The magnet-adsorbed cells were approximately 1 µm longer than the remaining cells at the same time points.

One unique feature of yeast mother cells is a bud scar left after every round of cell division. The number of bud scars can be used to demonstrate the number of cell divisions that mother cells have undergone. This study used Solophenyl Flavine 7GFE 500% for the visualization of bud scars (Figure 1b) [34]. Our measurement revealed that the bud scar counts of the magnet-adsorbed cells after 48 hours of culturing was nearly three times that of un-adsorbed cells (Fig. 1c). The larger cell size and increased bud scars demonstrated that the cells obtained by magnet possessed replicative senescent properties. Thus, this paper refers to the adsorbed cells as replicative senescent cells.

### Fragmented mitochondrial network dominates in the replicative senescent cells

**Figure 2:**
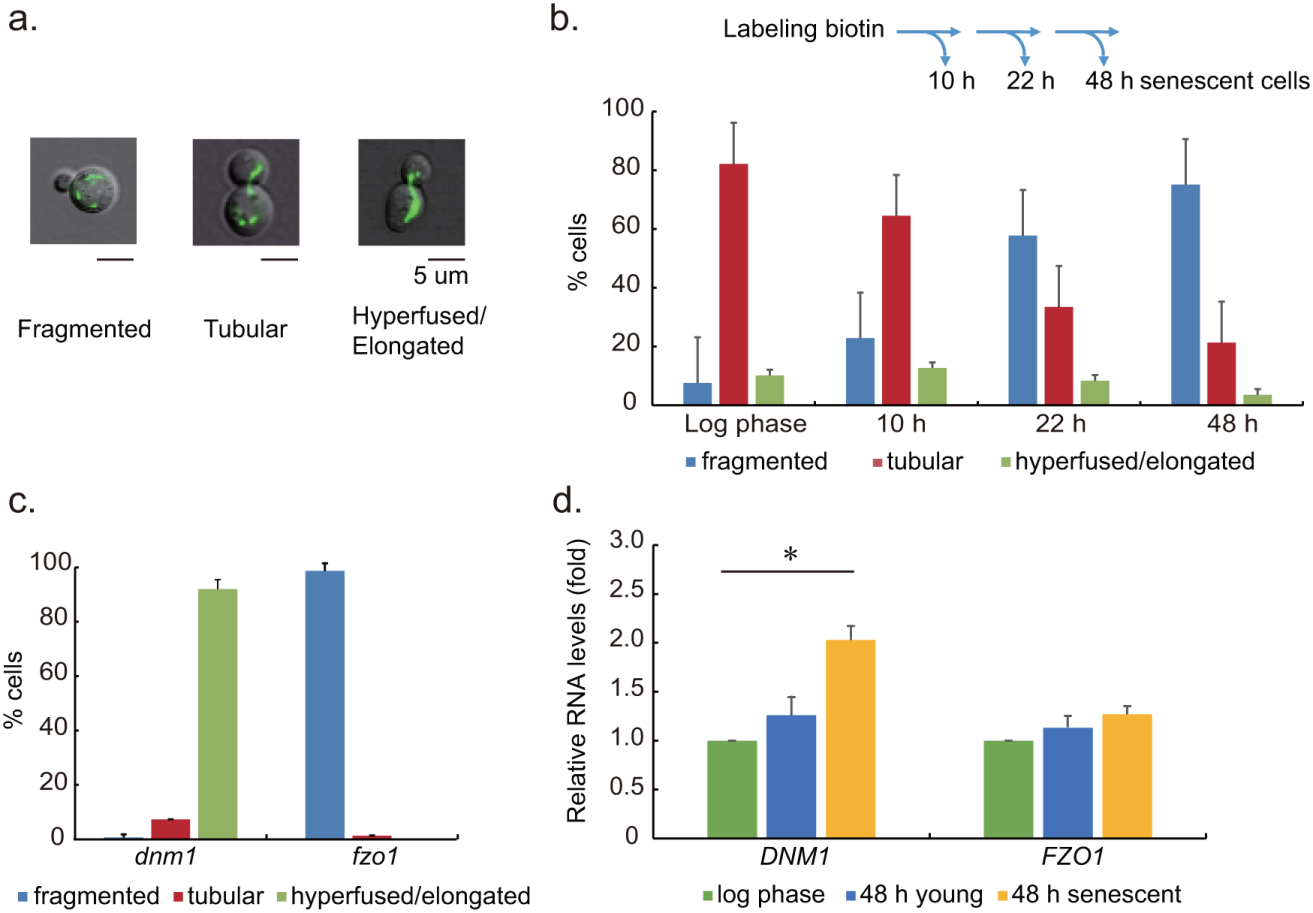
Mitochondrial network morphology and fusion/fission gene expression levels in replicative senescent cells. **a**. The mitochondria in yeast cells were labeled by green fluorescence protein (pVT100-MtGFP) for visualization. The morphology of mitochondrial network were classified into three categories: fragmented, tubular, and hyperfused/elongated. **b**. Senescent wild type cells were sorted at various time points after biotin labeling. Mitochondria network morphology of log phase cells and adsorbed senescent cells were classified. The ratio of senescent cells with fragmented mitochondria gradually increased with the duration of the culturing period. **c**. Senescent *dnm1* and *fzo1* cells at different time points were sorted and subjected for classifying mitochondrial network morphology. Almost none of the senescent cells from fission genes *DNM1* deleted strain presented a fragmented network. The same was true for senescent cells from the *FZO1* deleted strain, which presented all fragmented networks. **d**. Relative mRNA levels of *DNM1* and *FZO1* in wild type cells at the log phase of growth and senescence were measured by quantitative RT-PCR. *DNM1* levels in 48 h senescent cells were 2.5 fold higher than those in log phase and significantly higher than in young, not adsorbed cells (*: p<0.05).

Green fluorescent proteins targeted to mitochondria were used to visualize the shape of the overall network in this study. Fragmented, tubular and hyperfused/elongated were used to describe dotted, connected, and interconnected shape of mitochondrial network in cells (Fig. 2a). The classification of morphology was based on previous reports [8], [32], [33], and was judged by at least two individuals. At various time points after biotin labeling, samples were obtained using magnet sorting procedure for classification. The sorted senescent cells from different time points after biotin labeling were used to compared with cells in the log phase of growth (O.D. = 0.3). The ratio of senescent cells with fragmented mitochondrial network increased as the culturing time increased. The network morphology in most of the adsorbed cells remained tubular 10 hours after biotin labeling; however, more than half of the cells presented fragmented form after 22 hours. In replicative senescent cells obtained at 48 hour time point, 75% of them presented a fragmented mitochondrial network. In addition, we recorded the network morphology of cells that were not magnetically adsorbed remained as tubular, regardless of the time at which they were sampled. We had stained the 48 hr replicative senescent yeast cells with Annexin V and propidium iodide in order to detect whether the fragmented mitochondria we observed were correlated to early apoptosis. Our results indicated that the replicative senescent cells were not in early apoptosis (Table S1. The ratio of senescent cells stained by Annexin V and Propidium iodide).

Mitochondrial protein Fzo1 is crucial to mitochondrial fusion in yeast, while Dnm1 is the major protein associated with fission. This study sought to determine whether these conventional genes associated with fusion and fission play a role in the fragmentation of mitochondria in senescent cells. To achieve this, we constructed strains with *DNM1* and *FZO1* deletions. No fragmented network was observed in senescent *dnm1* cells (Fig. 2c). These results indicate that mitochondrial fragmentation in senescent cells required conventional fission protein Dnm1.

To elucidate the roles of fusion/fission genes in the mitochondrial fragmentation in senescent cells, we employed quantitative RT-PCR to examine the expression levels of *DNM1*and *FZO1* genes in wild type senescent yeast cells. Our results indicated that the *DNM1* expression level in 48 hour adsorbed senescent cells was nearly double that observed in log phase cells. No significant differences were observed in the expression of *FZO1* in senescent cells and log phase cells (Fig. 2d).

Changes in the morphology and gene expression profiles indicated differences in the mitochondria dynamics between senescent mother cells and young cells. Our results from fusion and fission gene deletion strains confirmed the process of mitochondrial fragmentation in senescent cells required conventional fission pathways.

### Replicative senescent cells have unique mitochondria assets

**Figure 3:**
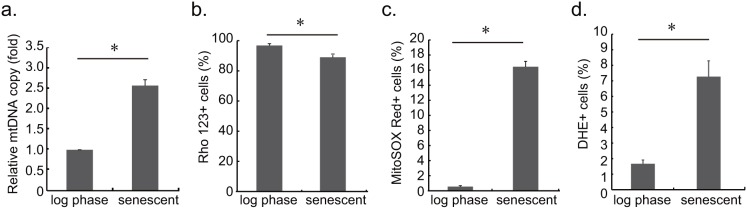
Mitochondrial DNA copy number, membrane potential and superoxide level in wild type cells. **a**. Relative copy numbers of the mtDNA in log phase and 48 h senescent cells were presented as fold changes. The relative mtDNA was normalized to genomic DNA by quantitative PCR. Our results indicate that the number of mtDNA copies in senescent cells was significantly higher than that obtained from log phase cells (p<0.05). **b**. Mitochondrial membrane potential was measured by Rhodamine 123 staining. Rho 123+ cells were determined by flow cytometry. The ratio of Rho 123+ cells in log phase cell was higher than 48 h senescent populations (p<0.05). **c**. Mitochondrial superoxide level was assayed by MitoSOX Red staining. MitoSOX Red+ cells were determined by comparing stained and unstained control cells. Log phase cells presented lower ratio of MitoSOX Red+ cells in the population compared to 48 h senescent cells. **d**. Intracellular superoxide level was examined by DHE staining. The ratio of DHE+ cells was significant higher in senescent yeast cells.

Mitochondria possess their own genes encoded specifically for oxidative phosphorylation (OXPHOS) and components required for their own transcriptional and translational machineries. Mitochondria dynamics is critical for the distribution and maintenance of mitochondrial DNA (mtDNA) [39]. Previous studies have demonstrated that fragmented mitochondria related to mtDNA loss [10], [40]. The large number of fragmented mitochondria in the senescent yeast cells in this study prompted an examination of the number of mtDNA copies by quantitative-PCR. To our surprise, we found senescent cells had 2.5 fold more mtDNA contents than those in log phase (Fig. 3a).

The quality and quantity of mtDNA are closely associated with oxidative phosphorylation and biogenesis potential. We questioned whether the relatively high number of mtDNA copies in senescent cells may be associated with changes in mitochondrial membrane potential and superoxide level. We examined the mitochondrial membrane potential using Rhodamine 123 (Rho 123) staining. Among wild type replicative senescent cells, 89.05% were Rhodamine 123+ cells. However, 96.78% of log phase cells were Rhodamine 123+ cells (Fig. 3b).

Since mitochondrial superoxide production is related to oxidative phosphorylation [41], staining senescent cells with MitoSOX Red was used to examine the of level mitochondrial superoxide. Flow cytometry results revealed significantly higher superoxide level in adsorbed senescent cells than in log phase cells (*p*<0.05) (Fig. 3c). In addition, Dihydroethidium (DHE) staining also demonstrated higher intracellular superoxide level in senescent cells (Fig. 3d). Thus, the fragmented mitochondria in senescent cells were linked to lower mitochondrial membrane potential, and to higher mtDNA copy numbers and superoxide level under our experimental conditions.

### Resveratrol reverts mitochondria fragmentation in senescent cells

**Figure 4:**
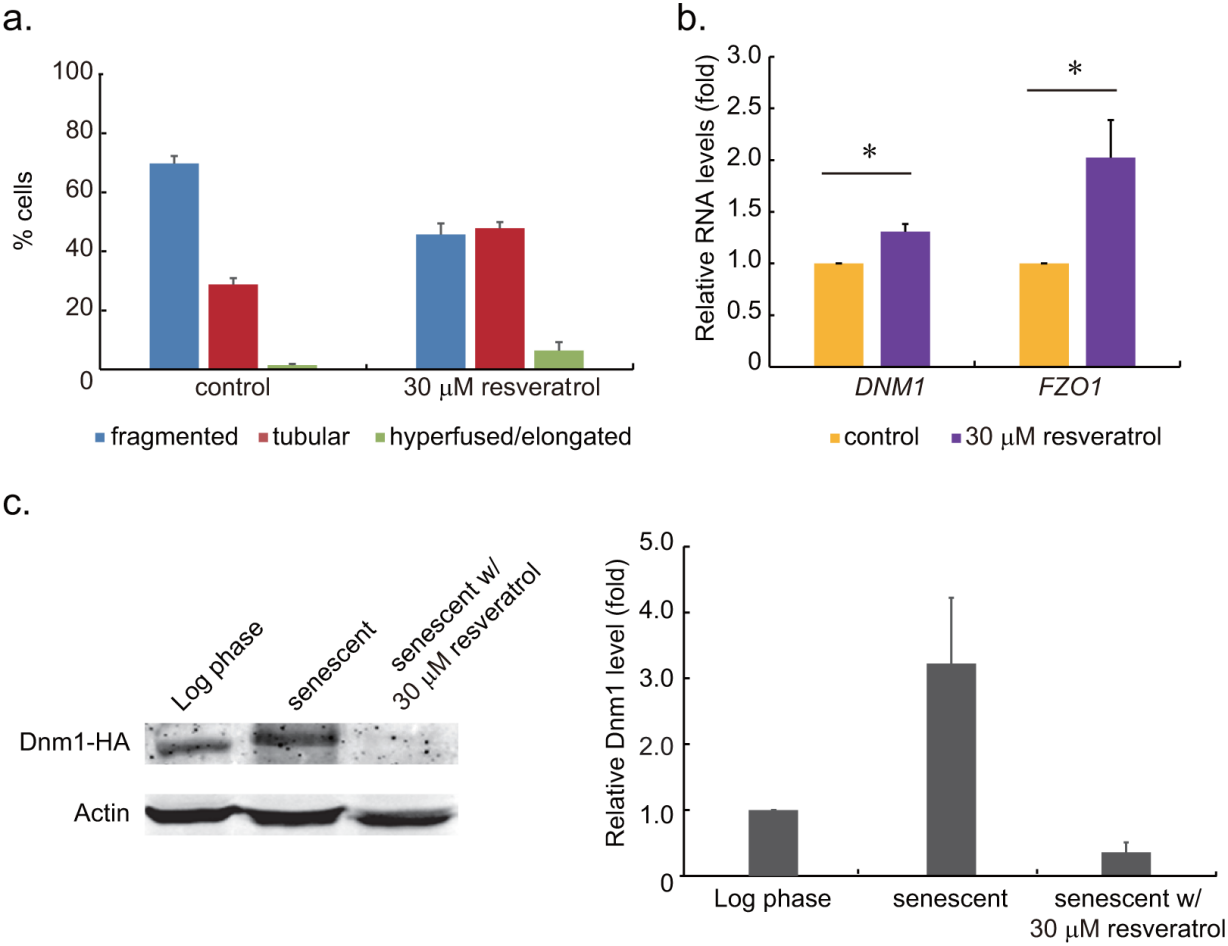
The effects of resveratrol on mitochondrial network morphology and fusion/fission gene expressions in replicative senescent cells. **a**. Mitochondria network morphology of control senescent cells and resveratrol-treated sample was classified. Resveratrol was added to the medium 14 hours after biotin labeling. The ratio of cells with fragmented mitochondria was 45% in resveratrol-treated sample, compared to 70% in the control group (p<0.05). **b**. Relative mRNA levels of *DNM1* and *FZO1* in senescent cells from the control sample and 30 µM resveratrol-treated senescent cells were measured by quantitative RT-PCR. Individual gene expression levels were normalized to that of the control cells (*: p<0.05). The *FZO1* gene level in resveratrol-treated senescent cells was approximately 2 times higher than that of the control cells. **c**. The Western blot and quantitation of Dnm1-HA in in log phase cells, senescent cells and senescent cells treated with 30 µM resveratrol. The relative Dnm1-HA protein levels were determined by Odyssey Imaging Systems. By normalizing to log phase cells, we found Dnm1 level in resveratrol treatment senescent cells was lower than untreated cells. The results demonstrated that Dnm1 level is correlated to fragmented status of mitochondrial network.

The elevated mitochondrial superoxide levels observed in our experiments is similar to those observed in earlier reports revealing higher levels of reactive oxygen species (ROS) in senescent cells [42], [43]. Resveratrol is a polyphenol, which scavenges free radicals and activates internal enzymatic antioxidants in cells. Thus, we treated senescent cells with resveratrol to elucidate its effects on the processes of fusion and fission. Mitochondrial morphology gradually becoming fragmented in senescent cells in our experiments. Based on our results, the numbers of cells with fragmented network equaled to the tubular ones should occur between 10 and 22 hours after biotin labeling. Taking this into consideration, we decided to add resveratrol (final concentration = 30 µM) to the culture medium at 14 hours after biotin labeling arbitrary. We let the resveratrol remained in the medium until senescent cells were sorted (48 hours after biotin labeling). We found that only 45% of senescent cells treated with resveratrol were with fragmented mitochondrial network (Fig. 4a). However, the ratio of senescent cells with fragmented network remained at 70% in the control sample. Treatments of 10, 50 and 100 µM resveratrol demonstrated proportional reduction in the number of cells with fragmented network, but we found higher concentrations of resveratrol treatments caused moderate reduction of the growth rate.

The reduction of senescent cells with fragmented mitochondrial network led us to examine the fusion/fission gene expression levels to clarify the effects of resveratrol. A significant 2 folds higher expression of fusion gene *FZO1* was found in the replicative senescent cells treated with resveratrol compared to control. Minor effects of *DNM1* expression was also observed (Fig. 4b). The differences of gene expression profiles between control and resveratrol-treated cells indicated involvement of resveratrol in the regulatory pathways of mitochondria dynamics. To further clarify the effects of fission gene expression level on mitochondria dynamics, we examined Dnm1 protein levels in log phase cells, senescent cells and senescent cells treated with resveratrol (Fig. 4c). We found that higher level of Dnm1 was correlated to fragmented mitochondria, and vice versa. Resveratrol-treated senescent cells were demonstrated to have lower Dnm1 level compared with untreated cells. These observations along with our findings of manipulating fusion/fission gene expression caused mitochondrial network morphology change (Figure S1. Overexpression of fission/fusion genes caused mitochondrial network morphology change in yeast cells.) supported the notion that mitochondria dynamics can be regulated through fusion/fission genes.

**Figure 5:**
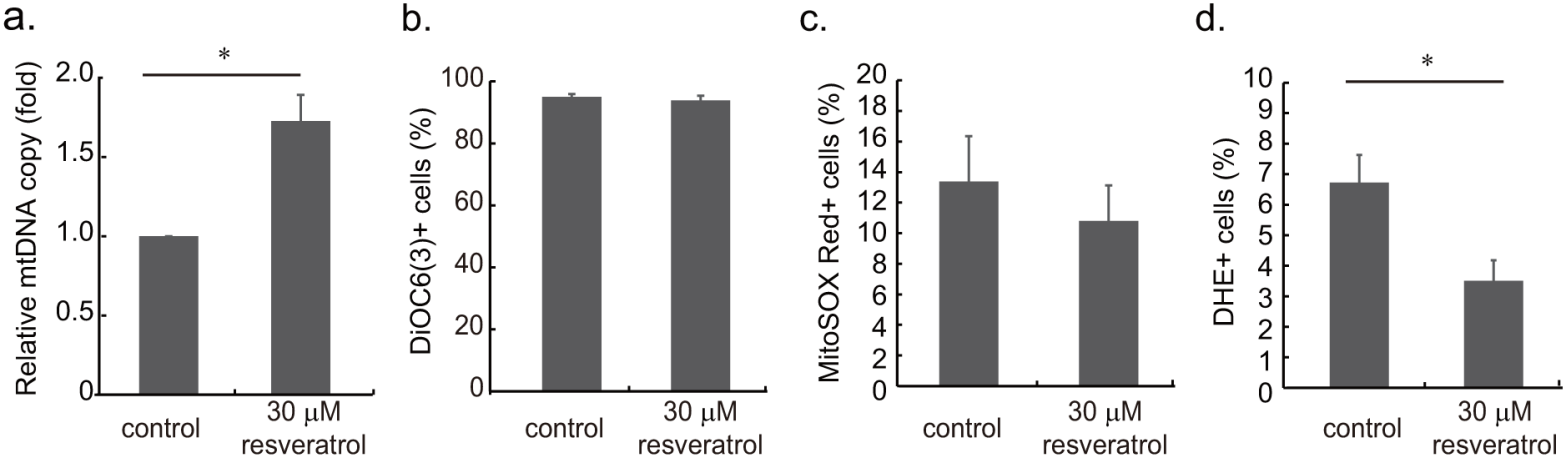
The effects of resveratrol on mtDNA, membrane potential and superoxide level in senescent cells. **a**. Relative mtDNA copy number of the control cells and senescent cells treated with resveratrol were measured by quantitative PCR. Cells treated with resveratrol had 1.73 folds higher of relative mtDNA contents than that of the control groups (*: p<0.05). **b**. Mitochondrial membrane potential was measured by DiOC_6_ (3) staining and flow cytometry. No significant differences were observed in the ratio of DiOC_6_ (3)+ cells between the control and resveratrol-treated cells. **c**. Mitochondrial superoxide level was assayed using MitoSOX Red staining. No significant difference was observed in the ratio of cells with MitoSOX Red+ between the control and resveratrol-treated senescent cells. **d**. Intracellular superoxide levels of control and resveratrol-treated cells were assayed by DHE staining. The ratio of DHE+ cells was lower in resveratrol-treated cells (*: p<0.05).

The reduction of senescent cells with fragmented mitochondrial network led us to examine the mtDNA copy number, membrane potential and superoxide level in resveratrol-treated cells. Based on quantitative PCR measurements, cells treated with resveratrol presented nearly double the number of mtDNA copies when compared with those of the control (Fig. 5a). No significant differences were observed between the control and treated samples with regard to mitochondrial membrane potential (Figs. 5b). We only detected minor effects of resveratrol on mitochondrial superoxide level, however, we did detect the reduction of intracellular superoxide level reduced by DHE staining (Fig. 5c and 5d).

## Discussion

This study collected replicative senescent yeast cells to examine mitochondria dynamics. We found that the ratio of cells with fragmented mitochondrial network increased with culturing duration. Resveratrol was shown to reduce the incidence of mitochondrial fragmentation and altered the expression profiles of genes related to mitochondrial fusion and fission.

### Mitochondrial fusion and fission is correlated to the status replicative senescence

We found replicative senescent yeast cells bear fragmented mitochondrial network with prolonged culturing period. However, contradictory results regarding the morphology of mitochondria in senescent cells has been reported. The formation of giant mitochondria caused by a reduction in hFis1 expression was reported in mammalian cells [44], [45]. A reduction in the ratio of mitochondria to total cell volume in senescent cells was observed by Rafelski et al. [46]. Hughes et al. also reported fragmented mitochondria in yeast mother cells sorted by mother enrichment program [47]. The balance of mitochondrial fusion and fission in senescent cells appear to be situated at the two ends of the spectrum in the two models; favors fusion in mammalian cells while favors fission in yeast cell. This may due to the fundamental differences of senescence in two models. The senescent replicative yeast cell in our experiments were estimated to have gone through 20–25 cell divisions. Considering a yeast mother cell may generate about 50 daughter cells, examining cells that have undergone more than 30 divisions surely will provide a better understanding of mitochondria dynamics in cases of extreme senescent condition. Nevertheless, our study as well as all previous works pointed out the differences of mitochondria dynamics between senescent and young cells. The cause for the shift from fusion toward fission, whether environmental or cellular factors, remains to be explored.

### Mitochondria dynamics in senescent cells relies on conventional fusion and fission genes

Our results indicate that *DNM1* is essential for the fragmentation of mitochondria in senescent cells, considering that deletion of *DNM1* prevented the mitochondria fragmentation and prominent expression in senescent yeast cells. The treatment of resveratrol reduced *DNM1* expression level and the ratio of senescent cells with fragmented mitochondria. Thus, the regulation of fusion and fission gene expression is regarded as a regulatory pathway for mitochondria dynamics [10], [48]–[50]. Data obtained in the current study support this notion.

The increase in the relative number of mtDNA copies in senescent yeast cells is similar to that observed in mammalian cells [51], [52]. One explanation for this may be that cells possess a mechanism to compensate for damage to mitochondrial DNA during aging processes. The minor mitochondrial membrane potential reduction in replicative senescent cells observed in our experiments supported the compensation theory. Without the compensation of increasing mtDNA for producing more components related to oxidative phosphorylation, lower mitochondrial activity and much fewer Rho 123+ cells were expected. However, our results did not suggest increased mtDNA copy number promoting mitochondria fusion since fewer replicative senescent cells with fragmented mitochondria were found in resveratrol-treated sample.

### Resveratrol mitigates mitochondria fragmentation in senescent cells by modulating gene expressions

Resveratrol treatment was shown to reduce the number of senescent cells with fragmented mitochondria as well as alterations of fusion and fission gene expression profiles. Higher *FZO1* levels may promote mitochondrial fusion and a subsequent reduction in the prevalence of mitochondrial fragmentation in senescent cells. It has been reported that mitochondrial hyperfusion is able to prevent further reduction of mitochondria numbers and compensate mitochondrial activity lost due to mutations of mtDNA [6], [53], [54]. The high number of mtDNA copies in resveratrol-treated cells may be a manifestation of its effects on mitochondrial biogenesis. Although the intracellular superoxide level was reduced in resveratrol-treated senescent cells, we noticed only minor effects of resveratrol on mitochondrial superoxide level and membrane potential. These results strongly suggest that resveratrol does not just act as an antioxidant. Rather, it appears to possess the ability to modulate fusion- and fission-related gene expression levels to maintain mitochondrial function. Thus, the superoxide production by mitochondria remained at a certain level in resveratrol-treated senescent cells.

Fragmented mitochondria has been associated with neurodegenerative diseases, such as Parkinson’s disease and Alzheimer’s disease, and these diseases are more prevalent in senior populations. In this study, we showed that resveratrol can modulate mitochondrial fusion and fission in senescent cells. Although more studies will be required to clarify the mechanisms underlying these effects, our results suggest that resveratrol is a good candidate for the treatment of diseases related to defects in mitochondria dynamics among an elderly population.

## Supporting Information

Figure S1 **Overexpression of fission/fusion genes caused mitochondrial network.** To examine the effects of overexpression of fission/fusion genes on mitochondria dynamics, *DNM1* and *FZO1* were constructed in pRS425 plasmids with ADH promoter, which constituently drives high levels of gene expression. The pRS425-ADH-DNM1 and pRS425-ADH-FZO1 were transformed into wild type yeast cells. The morphology of mitochondrial network in these fission/fusion gene overexpressed cells were classified. We found that most cells with overexpressed *DNM1* possessed fragmented mitochondria, while majority of cells with overexpressed *FZO1* have hyperfused/elongated mitochondria. morphology change in yeast cells.
(PDF)

Table S1 **The ratio of senescent cells stained by Annexin V and Propidium iodide.** Cells stained with the Annexin V-FITC and PI and were analyzed by flow cytometer. The 80 mM acetic acid-treated cells were served as apoptotic control groups. Based on our results, young and senescent groups from 48 hour sample had no significant increase of Annexin V (+) cells. Therefore, the senescent samples in our experiments were not in apoptosis.
(PDF)
