# Supplementary material for: Resveratrol Modulates Mitochondria Dynamics in Replicative Senescent Yeast Cells
Source: PLoS One. 2014 Aug 6;9(8):e104345. doi: 10.1371/journal.pone.0104345 (PMC4123921; doi:10.1371/journal.pone.0104345)
Supplement: Figure S1 — Overexpression of fission/fusion genes caused mitochondrial network. To examine the effects of overexpression of fission/fusion genes on mitochondria dynamics, DNM1 and FZO1 were constructed in pRS425 plasmids with ADH promoter, which constituently drives high levels of gene expression. The pRS425-ADH-DNM1 and pRS425-ADH-FZO1 were transformed into wild type yeast cells. The morphology of mitochondrial network in these fission/fusion gene overexpressed cells were classified. We found that most cells with overexpressed DNM1 possessed fragmented mitochondria, while majority of cells with overexpressed FZO1 have hyperfused/elongated mitochondria. morphology change in yeast cells. (PDF) [file pone.0104345.s001.pdf]

Figure S1. Overexpression of fission/fusion genes caused mitochondrial network morphology change in yeast cells.

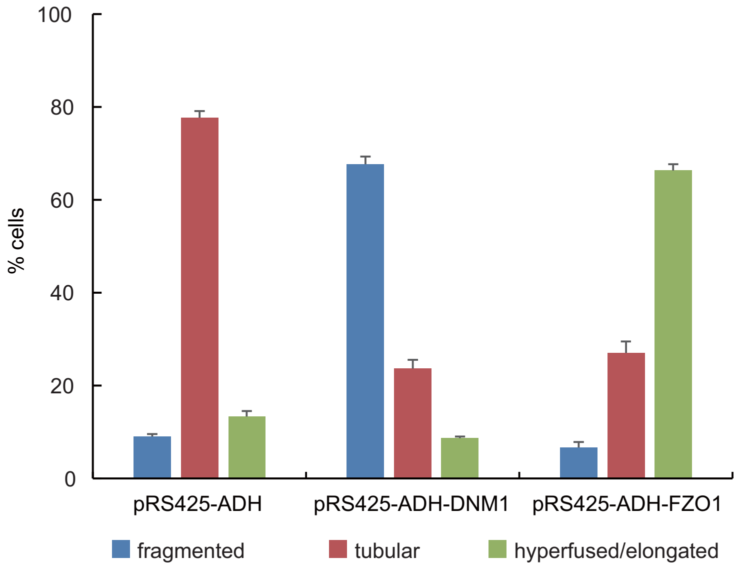

To examine the effects of overexpression of fission/fusion genes on mitochondria dynamics, *DNM1* and *FZO1* were constructed in pRS425 plasmids with ADH promoter, which constituently drives high levels of gene expression. The pRS425-ADH-DNM1 and pRS425-ADH-FZO1 were transformed into wild type yeast cells. The morphology of mitochondrial network in these fission/fusion gene overexpressed cells were classified. We found that most cells with overexpressed *DNM1* possessed fragmented mitochondria, while majority of cells with overexpressed *FZO1* have hyperfused/elongated mitochondria.
