# Supplementary material for: Resveratrol Modulates Mitochondria Dynamics in Replicative Senescent Yeast Cells
Source: PLoS One. 2014 Aug 6;9(8):e104345. doi: 10.1371/journal.pone.0104345 (PMC4123921; doi:10.1371/journal.pone.0104345)
Supplement: Table S1 — The ratio of senescent cells stained by Annexin V and Propidium iodide. Cells stained with the Annexin V-FITC and PI and were analyzed by flow cytometer. The 80 mM acetic acid-treated cells were served as apoptotic control groups. Based on our results, young and senescent groups from 48 hour sample had no significant increase of Annexin V (+) cells. Therefore, the senescent samples in our experiments were not in apoptosis. (PDF) [file pone.0104345.s002.pdf]

Table S1. The ratio of senescent cells stained by Annexin V and Propidium iodide.

	80 mM acetic acid	Log phase	48 hr young	48 hr senescent
**Annexin V (-)；PI (-)**	48.5%±1.95%	75.1%±6.8%	94.4%±6.5%	94.4%±3.0%
**Annexin V (+)；PI (-)**	37.8%±6.1%	20.9%±5.7%	6.9%±4.6%	5.0%±2.5%
**Annexin V (+)；PI (+)**	11.1%±5.6%	20.6%±7.9%	0.02%±0.03%	0.5%±0.3%
**Annexin V (-)；PI (+)**	17.1%±1.9%	6.3%±3.6%	0.4%±0.3%	0.3%±0.2%

Cells stained with the Annexin V-FITC and PI and were analyzed by flow cytometer. The 80 mM acetic acid-treated cells were served as apoptotic control groups. Based on our results, young and senescent groups from 48 hour sample had no significant increase of Annexin V (+) cells. Therefore, the senescent samples in our experiments were not in apoptosis.
